# Bounce-Based Aerobic Exercise Improves Postpartum Mood: A Large-Scale Pre–Post Study

**DOI:** 10.3390/brainsci16020133

**Published:** 2026-01-27

**Authors:** Shizuka Torashima, Sonoko Fujibayashi, Naoko Kishimoto, Mina Samukawa

**Affiliations:** 1 Department of Health Sciences, Faculty of Human Sciences, Osaka International University, Osaka 570-8555, Japan; n-kishimoto@oiu.jp; 2 Faculty of Psychology, Koshien University, Takarazuka 665-0006, Japan; fujibayashi@koshien.ac.jp; 3 Faculty of Health Sciences, Hokkaido University, Sapporo 060-0812, Japan; mina@hs.hokudai.ac.jp

**Keywords:** postpartum mental health, postpartum mood, perinatal mental health, aerobic exercise, bounce-based exercise, indoor exercise, physical activity, Profile of Mood States (POMS)

## Abstract

**Background:** Postpartum mental health challenges are increasingly recognized as a major public health concern, particularly during the early months after childbirth when emotional vulnerability is heightened. Although aerobic exercise is known to be associated with mood improvement, few large-scale studies have examined the acute psychological effects of a single exercise session in postpartum women, especially in contexts where environmental barriers restrict opportunities for physical activity. **Methods:** A total of 628 postpartum mothers (2–6 months postpartum) residing in a snowbound region of northern Japan participated in a single-session indoor bounce-based aerobic exercise program. Mood states were assessed immediately before and after the session using the validated Japanese short-form Profile of Mood States (POMS). Open-ended comments were also collected and analyzed thematically. **Results:** Paired analyses demonstrated significant acute improvements in key mood domains. Tension–Anxiety decreased (mean difference −7.91, 95% CI [−8.82, −7.00]; d = −0.68), and Anger–Hostility decreased (−4.61, 95% CI [−5.40, −3.82]; d = −0.45), while Vigor increased (11.82, 95% CI [10.47, 13.17]; d = 0.69) (all *p* < 0.001). In contrast, Depression–Dejection showed no significant change (−0.33, 95% CI [−1.30, 0.64]; *p* = 0.613). **Conclusions:** A single session of indoor bounce-based aerobic exercise was associated with immediate improvements in emotional well-being among postpartum women, particularly in anxiety-related and activation-related mood states. Because this exercise modality can be performed safely at home and is well suited to regions where outdoor physical activity is restricted, it may represent an accessible strategy for supporting postpartum mental health. Future research should examine longitudinal effects, controlled designs, and optimal program frequency to determine sustained benefits.

## 1. Introduction

The perinatal and postpartum periods represent critical phases in women’s lifelong physical and psychological health [1,2,3,4,5,6]. Although maternal and infant mortality rates in Japan have declined substantially due to advances in medical infrastructure, concerns regarding maternal emotional distress, parenting-related anxiety, and postpartum depression have become increasingly prominent in national reports and academic literature [2,7]. During the early postpartum months, rapid hormonal fluctuations, sleep deprivation, physical fatigue, and the continuous demands of infant care heighten vulnerability to mood disturbances and emotional dysregulation [8].

Recent national surveys have highlighted that, despite improvements in biomedical outcomes, psychosocial burdens among new mothers remain substantial [2,3]. Even within Japan’s highly developed obstetric care system, maternal deaths related to mental health conditions or postpartum complications continue to be reported [7]. Emotional instability during this period is associated with impaired bonding, breastfeeding challenges, and—in severe cases—postpartum depression (PPD) or a risk of maltreatment [8,9,10]. These concerns underscore the need for accessible, non-pharmacological strategies to support maternal mental well-being during early motherhood.

Aerobic exercise has long been recognized as an effective non-pharmacological approach for improving mood and mitigating psychological distress [11,12]. Research among pregnant women has consistently demonstrated reductions in anxiety, tension, and depressive tendencies following structured exercise programs [13,14,15]. However, empirical studies focusing specifically on postpartum women remain scarce. Existing interventions often involve multiple components—such as stretching, group support, or parent–child interaction—making it difficult to isolate the acute psychological effects of aerobic activity itself [16,17,18]. Moreover, most studies rely on small samples, limiting generalizability.

Environmental barriers further complicate postpartum mothers’ opportunities for physical activity. A unique contextual feature of Prefecture A, the setting of the present study, is its prolonged winter season, during which snow covers the ground for more than half the year. Such climatic conditions severely restrict outdoor exercise options such as walking or jogging [8,10]. For postpartum women—already constrained by infant care, limited mobility, and fatigue—indoor exercise modalities become particularly essential. In this context, safe and enjoyable activities that enable mothers to achieve moderate-intensity aerobic stimulation (5–6 METs) within the home environment hold substantial practical value [19,20,21].

Bounce-based aerobic exercise, performed on a balance ball [22] or soft flooring, has gained attention in Japan as a safe and engaging modality for both pregnancy and postpartum care [19,20,23].

The rhythmic vertical motion generates cardiovascular activation while minimizing joint impact, and physiological studies have noted beneficial effects on circulation, postural stabilization, and respiratory rhythm [19,20,21]. Despite its growing popularity and physiological plausibility, few studies have examined its acute psychological effects among postpartum mothers—particularly at scale [24].

To address these gaps, the present study conducted a large-scale pre–post evaluation of bounce-based aerobic exercise among 628 postpartum mothers. This makes the study one of the largest investigations to date examining immediate emotional responses to an isolated aerobic intervention in this population. Importantly, the present study was designed to isolate the acute psychological effects of aerobic movement itself, without incorporating additional components such as group support, parent–infant interaction, or stretching elements, which are commonly included in postpartum exercise programs.

The primary aim was to assess acute changes in mood states following a single bounce-based exercise session. A secondary aim was to explore the potential public health relevance of this modality for postpartum mental health support, particularly in snowbound regions where outdoor activity is limited [8,10].

## 2. Materials and Methods

### 2.1. Participants and Study Setting

A total of 628 postpartum mothers residing in Prefecture A, Japan, participated in this study. Participants were recruited through public health courses and community-based parenting programs offered at parenting support centers, public health centers, health promotion facilities, and childcare-support nonprofit organizations across six municipalities. All participants were between 2 and 6 months postpartum, reflecting natural enrollment patterns in local maternal–child health services [9,25].

Consistent with previous research in Japan, first-time mothers comprised the majority of the sample (92.5%, n = 579) [9,17]. The study period extended from July 2016 to January 2020. Prefecture A is characterized by a prolonged winter season, with snow covering the ground for more than half the year, markedly limiting opportunities for outdoor physical activity. This environmental context was an important consideration in the design and implementation of an indoor exercise intervention for postpartum women [26].

### 2.2. Study Design

The study employed a single-session pre–post intervention design to assess acute changes in mood states following a brief bout of bounce-based aerobic exercise. This design is suitable for detecting within-subject psychological responses occurring immediately after exercise, while minimizing confounding influences from long-term behavioral change, repeated training exposure, or social interaction effects. Although the absence of a control group limits causal inference, the large sample size and consistent directional changes across mood domains provide robust within-subject evidence of acute effects.

### 2.3. Aerobic Exercise Intervention (Bounce Exercise) [19,20,27,28]

The intervention consisted of a structured session of bounce-based aerobic exercise, an indoor modality previously utilized in Japanese maternal health programs [16,19]. Each session comprised

- Warm-up: 5 min of light rhythmic movement;
- Main exercise: 15–20 min of bounce-based aerobic activity;
- Cool-down: 5 min of relaxation and controlled breathing.

All sessions were conducted indoors to ensure safe participation regardless of weather conditions [10]. Exercise intensity corresponded to approximately 5–6 METs, representing a moderate aerobic load appropriate for postpartum women [20,21]. Intensity was guided by instructor demonstration and participant self-monitoring of perceived exertion, consistent with previously validated postpartum exercise protocols in Japan.

Physiological studies suggest that rhythmic vertical bouncing promotes venous return, stabilizes breathing patterns, and activates core and postural musculature—mechanisms that may contribute to both physiological and emotional benefits [19,20].

### 2.4. Measurement of Mood and Emotional States (POMS)

Mood states were assessed using the validated Japanese short-form Profile of Mood States (POMS; Kaneko Shobo, Tokyo, Japan), which comprises 30 items across six subscales [29]:

1. Tension–Anxiety;
2. Depression–Dejection;
3. Anger–Hostility;
4. Vigor;
5. Fatigue;
6. Confusion.

Participants rated each item on a 5-point scale. Scores were calculated using age-adjusted norms and standardized scoring procedures [29]. Mothers completed the POMS immediately before and immediately after the exercise session to capture acute mood changes [11,24].

### 2.5. Open-Ended Subjective Impressions

Following the second POMS assessment, participants were invited to provide optional written comments describing emotional or physical changes they experienced during the session. A total of 604 mothers provided comments. Responses were reviewed and categorized thematically. Two trained coders independently grouped comments into categories; discrepancies were resolved through discussion to ensure reliability of classification [30,31].

Initial agreement between coders was high, and all discrepancies were resolved through discussion to reach consensus; formal inter-rater reliability coefficients were not calculated due to the exploratory nature of the thematic analysis. Of the total sample (N = 628), 604 participants provided open-ended comments. The remaining participants did not provide comments primarily due to time constraints or personal preference, rather than exclusion criteria.

### 2.6. Statistical Analysis

Pre–post differences in POMS subscale scores were analyzed using paired *t*-tests. Mean differences, 95% confidence intervals, and Cohen’s d for paired samples were calculated. Because six POMS subscales were analyzed, Bonferroni-adjusted significance levels were additionally examined as a sensitivity analysis to assess robustness against multiple comparisons; the primary findings remained statistically significant after correction. All statistical analyses were conducted using Microsoft Excel 2013 (Microsoft Corporation, Redmond, WA, USA), and statistical significance was defined as *p* < 0.05 (two-tailed).

### 2.7. Ethical Considerations

All procedures adhered to the Hokkaido University of Education Research Ethics Code (Approval Number: 2016120006). Participants were informed about the study purpose, voluntary participation, and confidentiality. Written informed consent was obtained from all participants prior to data collection [3].

## 3. Results

### 3.1. Changes in POMS Scores Before and After Exercise

**Figure 1:**
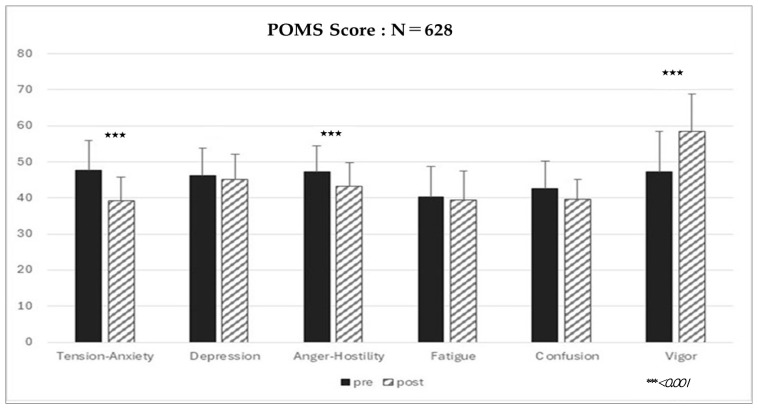
POMS Score (N = 628).

Significant improvements were observed across several POMS subscales following the exercise session (Figure 1, Table 1).

Tension–Anxiety decreased from 47.7 ± 8.2 to 39.3 ± 6.6 (*p* < 0.001), and Anger–Hostility decreased from 47.2 ± 7.21 to 43.2 ± 4.98 (*p* < 0.05). Depression–Dejection showed a small, non-significant decrease (from 46.2 ± 7.56 to 45.1 ± 7.01). Vigor increased markedly from 47.2 ± 11.3 to 58.5 ± 10.17 (*p* < 0.001). Fatigue and Confusion demonstrated small, non-significant decreases. To provide a clearer overview of pre–post changes, all subscale scores, associated t-values, *p*-values, and effect sizes are summarized in Table 1. As shown, Tension–Anxiety and Vigor exhibited the largest changes, while Anger–Hostility demonstrated a medium-sized improvement. Depression–Dejection showed only a small, non-significant change, consistent with the overall pattern observed in the present study.

Paired t-test analyses revealed significant reductions in Tension–Anxiety (mean difference = −7.91, 95% CI [−8.82, −7.00], t(627) = −17.02, *p* < 0.001) and Anger–Hostility (mean difference = −4.61, 95% CI [−5.40, −3.82], t(627) = −10.18, *p* < 0.001), as well as a significant increase in Vigor (mean difference = 11.82, 95% CI [10.47, 13.17], t(627) = 15.88, *p* < 0.001). In contrast, Depression–Dejection showed no significant change (mean difference = −0.33, 95% CI [−1.30, 0.64], t(627) = −0.88, *p* = 0.613). All significant results remained robust after Bonferroni correction for multiple comparisons.

The observed pattern suggests that a single session of moderate-intensity bounce-based aerobic exercise can rapidly reduce tension- and irritation-related mood states while simultaneously enhancing positive activation [11,12]. These summarized results in Table 1 correspond closely with the visual pattern shown in Figure 1, indicating consistent pre–post improvements across key mood domains [11].

### 3.2. Subjective Impressions from Open-Ended Comments

A total of 604 mothers (96%) submitted optional written comments describing emotional and physical sensations following the exercise session. The majority of comments were positive and aligned with the quantitative findings.

The most frequently reported themes were

1. “I felt refreshed both physically and mentally.” (88%)
2. “Fatigue and heaviness disappeared.” (72%)
3. “My stress was relieved.” (61%)
4. “It provided a good change of mood.” (54%)
5. “It was enjoyable and fun (like being on a trampoline).” (29%)

**Figure 2:**
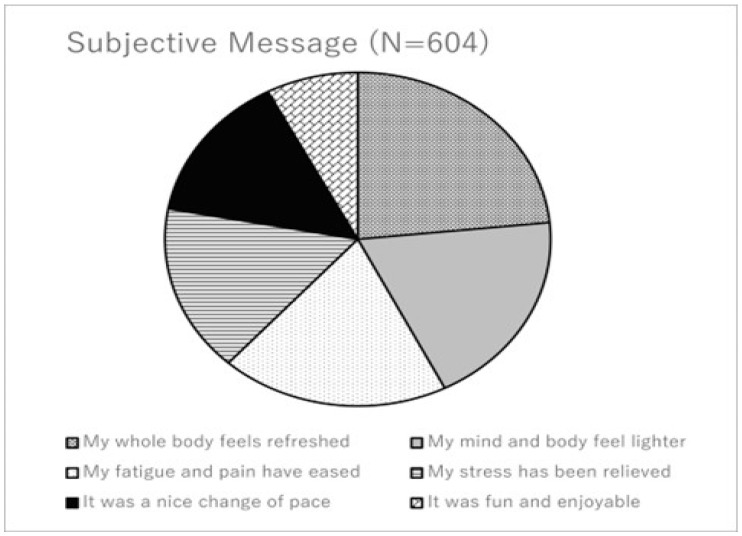
Distribution of subjective comments categorized into five themes (N = 604).

These subjective impressions reinforce the quantitative improvements and suggest high acceptability, immediate perceived benefit, and positive emotional engagement with the exercise modality [11,30,31] (Figure 2).

## 4. Discussion

This study demonstrated that a brief, single session of indoor bounce-based aerobic exercise was associated with immediate improvements in emotional well-being among postpartum mothers. With a sample size of 628 participants, this investigation represents one of the largest single-session studies to date examining acute psychological responses to aerobic exercise in postpartum populations [24]. The large sample size enhances the robustness of the findings and provides valuable real-world evidence relevant to maternal health promotion.

The significant reductions observed in Tension–Anxiety and Anger–Hostility, together with the marked increase in Vigor, indicate that moderate-intensity rhythmic aerobic activity can exert acute effects on mood regulation. These findings are consistent with previous research showing that aerobic exercise is associated with reduced stress and negative affect, as well as enhanced positive activation and energy levels [11,12]. Effect size analyses further support this pattern, with large effects observed for Tension–Anxiety and Vigor and a medium effect for Anger–Hostility. The consistency of these effects across multiple mood domains underscores the robustness of the observed acute psychological response to bounce-based aerobic exercise [16,17,18].

From a physiological perspective, rhythmic vertical bouncing may promote venous return, enhance oxygenation, and facilitate respiratory entrainment—mechanisms that may contribute to increased alertness and reduced tension [8,10,32]. The moderate exercise intensity (approximately 5–6 METs) is likely appropriate for postpartum women, who often experience concurrent physical fatigue and psychological strain [19,20,33]. Importantly, the single-component design of the present intervention—isolating aerobic movement without additional social, stretching, or parent–infant interaction components—represents a methodological strength that allows clearer attribution of observed mood changes to rhythmic aerobic activity itself.

A notable finding of this study was the minimal acute change observed in Depression–Dejection scores. This pattern aligns with existing evidence suggesting that depressive mood states typically require repeated exercise exposure [34] or longer-term interventions to demonstrate measurable improvement. Acute exercise appears to exert more immediate effects on anxiety-related and activation-related mood dimensions, whereas depressive symptoms may respond more gradually [19,20]. Thus, the present results are coherent with established exercise–mood mechanisms [24].

Environmental context is another critical factor in interpreting these findings. Prefecture A is characterized by prolonged winter conditions, during which snow substantially limits outdoor physical activity for extended periods. Environmental barriers such as severe weather, limited access to safe walking spaces, and childcare responsibilities may further restrict postpartum women’s opportunities for exercise. In this context, indoor exercise modalities that enable moderate aerobic intensity without leaving home are particularly valuable [11,33]. Bounce-based exercise is accessible, low-impact, and requires minimal space, aligning well with the needs of postpartum women living in environmentally constrained settings.

Qualitative data further reinforced the quantitative findings. Subjective impressions provided by more than 600 mothers frequently described feelings of refreshment, lightness, and reduced stress, indicating high acceptability and perceived emotional benefit [30,31]. Enjoyment and perceived ease are known predictors of exercise adherence, suggesting that bounce-based exercise may represent a sustainable option when integrated into community-based postpartum health programs.

Although the present study focused on acute mood responses, these immediate emotional improvements may have implications for daily functioning and overall maternal well-being [35]. However, parenting outcomes and clinical postpartum depression were not directly assessed. Future research should therefore examine longitudinal effects, dose–response relationships, and the impact of repeated bounce-based exercise using controlled designs and validated screening or diagnostic tools for postpartum depression [8,9,10,18].

## 5. Conclusions

In conclusion, a single session of indoor bounce-based aerobic exercise was associated with acute improvements in emotional well-being among postpartum mothers. Significant reductions in tension- and irritation-related mood states, together with a marked increase in vigor, suggest that moderate-intensity rhythmic aerobic activity can produce meaningful immediate psychological benefits.

Because this exercise modality is safe, enjoyable, and feasible to perform at home—even in snowbound regions—it represents a promising and accessible strategy for postpartum mental health promotion in community settings. Nevertheless, the present findings reflect short-term effects following a single exercise session, and further research is required to determine whether repeated or long-term participation leads to sustained improvements in mood or clinically relevant outcomes.

## 6. Limitations

This study has several limitations. First, the single-session pre–post design did not include a control group, which limits causal inference and precludes attribution of the observed effects solely to the intervention. Second, Depression–Dejection did not show a significant acute change, and the study did not assess longer-term outcomes or clinical diagnoses of postpartum depression, limiting conclusions regarding sustained or clinical effects. Third, mood responses may have been influenced by contextual or interpersonal factors inherent to group-based exercise sessions, such as instructor guidance or group atmosphere. Finally, all data were collected in a snowbound region of Japan, which may limit generalizability to other geographic or climatic contexts.

Future research should incorporate follow-up assessments, randomized controlled designs, and comparisons with other exercise modalities to clarify causal mechanisms, optimal program structures, and long-term mental health benefits.

Although open-ended comments were obtained from 604 of the 628 participants, the remaining mothers did not provide comments mainly due to time constraints or personal preference. Therefore, a potential response bias cannot be completely ruled out; however, the qualitative data were used to complement, rather than replace, the quantitative findings.

## Figures and Tables

**Table 1:** Changes in POMS subscale scores before and after bounce-based aerobic exercise (N = 628).

Subscale	Mean Difference (Post-Pre)	SD of Difference	t-Value	*p*-Value	95% CI
Tension–Anxiety	−7.91	11.66	−17.02	<0.001	[−8.82, −7.00]
Anger–Hostility	−4.61	10.14	−10.18	<0.001	[−5.40, −3.82]
Depression–Dejection	−0.33	12.34	−0.88	0.613	[−1.30, 0.64]
Vigor	11.82	17.18	15.88	<0.001	[10.47, 13.17]

## Data Availability

The data presented in this study are available on request from the corresponding author due to ethical restrictions and the protection of participants’ privacy.
